# Dissociating Value Representation and Inhibition of Inappropriate Affective Response during Reversal Learning in the Ventromedial Prefrontal Cortex^1^,^2^,^3^

**DOI:** 10.1523/ENEURO.0072-15.2015

**Published:** 2016-01-04

**Authors:** Zhihao Zhang (张之昊), Avi Mendelsohn, Kirk F. Manson, Daniela Schiller, Ifat Levy

**Affiliations:** 1 Interdepartmental Neuroscience Program, Yale University, New Haven, Connecticut 06520; 2 Section of Comparative Medicine, Yale School of Medicine, New Haven, Connecticut 06520; 3 Department of Neuroscience, Yale School of Medicine, New Haven, Connecticut 06520; 4 Sagol Department of Neurobiology, University of Haifa, Haifa, Israel 3498838; 5 Department of Psychiatry, Department of Neuroscience and Friedman Brain Institute, Icahn School of Medicine at Mount Sinai, New York, New York 10029

**Keywords:** conditioning, fMRI, human, reward-learning, valuation

## Abstract

Decision-making studies have implicated the ventromedial prefrontal cortex (vmPFC) in tracking the value of rewards and punishments. At the same time, fear-learning studies have pointed to a role of the same area in updating previously learned cue–outcome associations. To disentangle these accounts, we used a reward reversal-learning paradigm in a functional magnetic resonance imaging study in 18 human participants. Participants first learned that one of two colored squares (color A) was associated with monetary reward, whereas the other (color B) was not, and then had to learn that these contingencies reversed. Consistent with value representation, activity of a dorsal region of vmPFC was positively correlated with reward magnitude. Conversely, a more ventral region of vmPFC responded more to color A than to color B after contingency reversal, compatible with a role of inhibiting the previously learned response that was no longer appropriate. Moreover, the response strength was correlated with subjects’ behavioral learning strength. Our findings provide direct evidence for the spatial dissociation of value representation and affective response inhibition in the vmPFC.

## Significance Statement

Numerous studies have implicated the ventromedial prefrontal cortex (vmPFC) in value encoding, forming the basis for decision-making. A separate line of research has associated the same region with a critical role in negative-affect regulation. Are these two distinct functions of the vmPFC or simply different manifestations of the same process? Using a task that requires both value representation and affect regulation, yet enables to distinguish between the neural correlates associated with each, we found that these two processes are localized in different subregions of the vmPFC. Such findings bridge two previously disconnected branches of cognitive neuroscience research and advance our understanding of the functional organization of the vmPFC.

## Introduction

Decision neuroscience has identified the ventromedial prefrontal cortex (vmPFC) as a constituent of a “valuation system” in the brain. Together with the ventral striatum, this region appears to encode a value signal that guides action selection and choice (Montague and Berns, 2002; O'Doherty, 2004; Knutson and Cooper, 2005; Rangel et al., 2008; Kable and Glimcher, 2009; Peters and Büchel, 2010; Grabenhorst and Rolls, 2011; Levy and Glimcher, 2012; Roy et al., 2012). A recent meta-analysis (Bartra et al., 2013) characterized the response profile of the vmPFC during decision-making tasks by examining 206 functional magnetic resonance imaging (fMRI) studies, which measure blood oxygenation level-dependent (BOLD) signal. According to this meta-analysis, the vmPFC BOLD signal scales positively with reward value at both the time of decision and when the reward is delivered, thereby encoding the value of both primary and secondary incentives (eg, food and money, respectively).

The neuroscience of punishment-driven learning has reached a different conclusion. Studies using classical fear conditioning consistently find that the vmPFC BOLD signal correlates with the updating of a learned fear response (Phelps et al., 2004; Milad et al., 2005b; Kalisch et al., 2006; Milad et al., 2007a; Delgado et al., 2008; Schiller et al., 2008; Milad and Quirk, 2012; Schiller et al., 2013). This finding repeats in tasks using various strategies for inhibiting the fear response to a stimulus that was previously paired with an aversive outcome (Schiller and Delgado, 2010). More generally, a meta-analysis (Diekhof et al., 2011) has shown that the vmPFC is central to the downregulation of negative affect independent of experimental design. This line of human research builds on a large body of evidence from animal research, describing the detailed neural circuitry in which projections from the rat vmPFC to the amygdala modulate conditioned threat responses (Rolls, 2004; Myers and Davis, 2007; Sotres-Bayon et al., 2007; Quirk and Mueller, 2008; Delamater and Westbrook, 2014). Importantly, the bulk of the evidence suggests that the vmPFC is involved in the expression of learning, rather than in driving that learning. For example, damage to the vmPFC only affected the retention and delayed expression of extinction learning (Quirk et al., 2000), and vmPFC neurons only responded to a conditioned stimulus during a delayed test of extinction (Milad and Quirk, 2002). Similarly, in a fear-reversal paradigm, the vmPFC responded to the stimulus that used to predict shock and ceased to do so, but not immediately after the switch in contingencies (Schiller et al., 2008).

**Figure 1.**
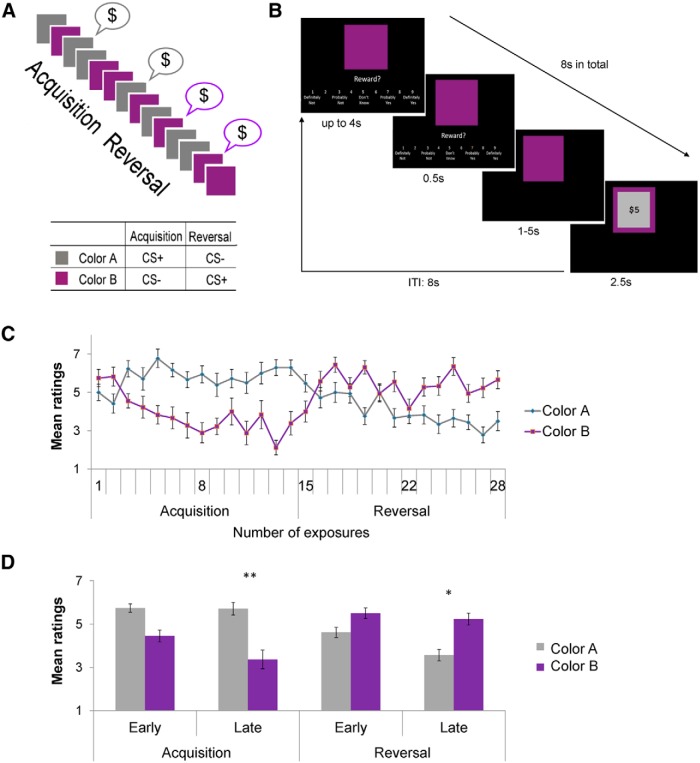
Behavioral task and performance. ***A***, Overall timeline. The acquisition stage consisted of presentations of two colored squares on a partial reinforcement schedule. Color A was associated with reward on about a third of the trials (CS+), whereas color B was not (CS−). In the reversal stage the reward contingencies were switched, such that color B was now paired with reward (new CS+) and color A was not (new CS−). The first trial in which color B was followed by a reward marked the beginning of the reversal stage. Gray and purple were the actual colors used in the experiment, and the assignment of colors to color A and color B was counterbalanced across participants. ***B***, Within-trial timeline. Stimuli were presented in pseudorandom order together with a rating scale for a maximum of 4 s. After the participant provided the rating, the appropriate number was highlighted on the screen for 0.5 s. After a variable delay period that lasted between 1 and 5 s (the actual duration depended on the time that the participant took to provide the expectancy rating, keeping the duration of the entire trial constant at 8 s), the outcome was presented for 2.5 s. On one-third of the CS+ trials, a reward image was then superimposed on the colored square, indicating the reward received on that trial. On the remaining CS+ trials and all CS− trials, no reward image was shown. Trials were separated by an 8 s intertrial interval. Before starting the task, it was made clear to the participants that at the end of the experiment they would receive the accumulated money rewards they saw during the experiment. ***C***, Reward expectancy ratings throughout the task. Mean reward expectancy ratings to the two stimuli are plotted as a function of the number of exposures to each. Error bars represent SEM. Only non-reinforced trials were included. Participants successfully learned the changing reward contingencies, as shown by higher ratings to color A by the end of the acquisition stage and the reverse trend by the end of the reversal stage. ***D***, Reward expectancy ratings in four phases of the task. The acquisition and reversal stages were divided into early (the first half) and late (the second half) phases. Error bars, SEM. A three-way repeated-measures ANOVA with factors including stimulus (colors A and B), stage (acquisition, reversal), and phase (early, late) revealed a significant stimulus × stage × phase interaction (*F*_(1,17)_ = 8.951, *p* < 0.01). Asterisks indicate the significance of post-hoc tests (Bonferroni correction applied) comparing the difference in reward expectancy ratings between CS+ and CS− at each stage. *p < 0.05; **p < 0.01.

The two possible roles attributed to the vmPFC, signaling reward value and inhibiting a learned aversive response, are not necessarily contradictory. The omission of an aversive outcome could be represented as a positive event; so, greater BOLD signal to a stimulus that used to predict punishment and became a safety signal is consistent with either account. How could we tell the two functions apart? We followed the design of a fear reversal learning study (Schiller et al., 2008) with a key modification: we replaced aversive outcomes with appetitive ones. The experimental procedure included an acquisition stage immediately followed by a reversal stage (Fig. 1). During acquisition, one stimulus (a colored square) coterminated with monetary reward on approximately one-third of the trials (conditioned stimulus, CS+; color A), and another stimulus (CS−, color B) did not terminate with reward. The reversal stage began when the reinforcement contingencies switched; color B (new CS+) now coterminated with reward and color A did not (new CS−).

The critical event occurs when the CS+ (color A) ceases to predict the reward during the reversal stage. Unlike aversive reversal (Schiller et al., 2008), the two different accounts of the vmPFC now lead to opposite predictions. If the vmPFC represents an inhibitory signal, we would predict increased vmPFC BOLD responses to color A during reversal, because the previously learned reward response is no longer appropriate and should be suppressed. If the vmPFC BOLD signal positively correlates with reward value, however, we would expect decreased vmPFC responses to color A because it is no longer accompanied by the monetary outcomes, and is thus less rewarding. In this case, the vmPFC signal would increase whenever reward is delivered.

Given the well-established anatomical and functional heterogeneity of the vmPFC (Ongür and Price, 2000), value representation and regulation of negative affect may be localized in distinct parts within the vmPFC. A closer examination of the coordinates reported by the two corresponding meta-analyses indeed suggests a potential spatial segregation between these two functions (Bartra et al., 2013, value representation: *X* = −1, *Y* = 46, *Z* = −7; Diekhof et al., 2011, regulation of negative affect: *X* = 0, *Y* = 40, *Z* = −18; coordinates are in Montreal Neurological Institute coordinate space). Therefore, we hypothesized that separate subregions within the vmPFC simultaneously encode reward value and response inhibition.

## Materials and Methods

### Participants

Twenty-two healthy right-handed volunteers were recruited for the fMRI task. Four participants had excessive head motion during the fMRI scan and were excluded from further analysis. The final sample included eighteen healthy right-handed volunteers (7 males) between 19 and 34 years of age (mean 24.6 ± 4.9 SD). The experiment was approved by the Yale University Human Investigation Committee. All participants gave informed consent and were paid for their participation.

### Behavioral paradigm

An appetitive reversal learning task was used (Fig. 1A), with two colored squares as conditioned stimuli (CS). The use of a discrimination procedure allowed us to detect differences in the learned predictive properties of these stimuli. The unconditioned stimulus (US) was $5 (8 trials) or $10 (6 trials). A standard script for the instructions was strictly followed, and participants were instructed to try to figure out the relationship between the colored squares and the rewards. No mention was made of two stages (see below) or of a reversal of contingencies.

In the first stage, acquisition, one color (color A) was paired with the US on one-third of the trials (CS+), and the other (color B) was never paired with the US (CS−). The purpose of using partial reinforcement was to make learning nontrivial and to slow acquisition and reversal. This allowed us to examine the early and late phases in each stage and the gradual development of appetitive learning and its reversal. In the second stage, reversal, reward contingencies was reversed, such that color B was now paired with the US on approximately one-third of the trials (new CS+) and color A was not paired with the US (new CS−). The order of the different trial types was pseudorandomized (no consecutive reinforced trials and no more than 2 consecutive trials of each kind), and the designation of colors into CS+ and CS− was counterbalanced across participants. Two pseudorandom trial sequences were used, and participants were randomly assigned to one of them. During both acquisition and reversal, there were 14 presentations of each of the CSs, intermixed with seven additional presentations of the CS+ that coterminated with the US. This allowed us to include equal numbers of CS+ and CS− trials in subsequent analyses, excluding CS+ trials coterminating with the US, in which BOLD responses to the CS may have been contaminated by the response to the monetary reward. Reversal immediately followed acquisition, and the transition between the stages was unsignaled. To gauge the development of learning over time, the first and second halves of both stages were defined as early and late phases, respectively. Thus, the entire paradigm consisted of four phases, early acquisition, late acquisition, early reversal, and late reversal. All cues and outcomes were programmed into the script in advance, and the outcomes did not depend on the responses made by the participant.

Participants’ task was to indicate, on a 1–9 scale, the degree to which they expected to get a reward on the following screen. The scale appeared on the screen together with each CS, with verbal descriptions of the options (Fig. 1*B*). For example, 1, 5, and 9 corresponded to “definitely not”, “don’t know”, and “definitely yes”, respectively. Participants had up to 4 s to respond by pressing one of two buttons to decrease or increase the number along the 1–9 scale, and a third button to confirm the answer. The chosen number was highlighted for 0.5 s. Afterward, the scale and the notes disappeared, whereas the CS remained on the screen for the remainder of 5.5 s. If it was a US trial, an image of the monetary reward was superimposed on the colored square for 2.5 s; otherwise, only the colored square was on the screen for the same amount of time. The length of each trial was held constant at 8 s regardless of the participant’s response time, and there was an intertrial interval of 8 s.

Before the experimental session, participants underwent a brief instruction session and four practice trials. To avoid interference with learning in the main task, there were no rewards in the practice trials, which participants knew in advance. The colors of the CSs in the practice trials were also different from those of the CSs in the main task. It was emphasized to the participants that at the end of the experiment, they would receive the accumulated amount of all the money that they saw during the experiment. This resulted in a total of $100, which was added to the show-up fee.

### Neuroimaging acquisition and analysis

Participants were scanned in a 3T Siemens Trio scanner, using a 12-channel receiver array head coil. High-resolution, T1-weighted anatomical images were collected for each subject using an MPRAGE sequence at a 1 × 1×1 mm resolution. Functional data were collected using a standard EPI sequence (TR = 2 s, TE = 20 ms, 40 near axial slices, 3 × 3×3 mm, 64 × 64 matrix in a 192 × 192 mm FOV) and local shimming to the field-of-view. Analysis of the imaging data were conducted using BrainVoyager QX, NeuroElf software packages (http://www.neuroelf.net) and additional in-house MATLAB functions. Functional imaging data preprocessing included discarding the first eight volumes, motion correction, slice scan time correction (using sinc interpolation), spatial smoothing using a three-dimensional Gaussian filter (6 mm FWHM), voxelwise linear detrending, and high-pass filtering of frequencies above three cycles per time course. Four participants (of the initial 22) with motion >2 mm were not included in the analysis. Structural and functional data of each participant were then transformed to standard Talairach stereotaxic space (Talairach and Tournoux, 1988).

Statistical analysis was based on a general linear model. Each trial was divided into three periods: (1) the stimulus onset period at the beginning (0–2 s) of a trial, (2) the delay period (2–6 s), and (3) the outcome period at the end of a trial (6–8 s). CS onset was modeled by a binary regressor and a parametric regressor modulated by the reward expectancy ratings that each participant provided. For the delay period, separate binary predictors were constructed for each trial type (colors A and B) at each of four phases, early and late acquisition, and early and late reversal. Outcome phase was modeled by separate binary predictors for rewarded and non-rewarded trials combining both colors. Reward outcome was further modeled by a parametric regressor modulated by reward magnitude. Ratings and reward magnitudes were demeaned prior to creating the parametric regressors. Six motion parameters were included as regressors of no interest. All regressors were convolved with a standard canonical hemodynamic response function. Activation during intertrial intervals served as baseline.

In a whole-brain single-subject analysis, the model was independently fit to the activity time course of each voxel, yielding 13 coefficients for each participant (stimulus onset, reward expectancy ratings, 8 delay period regressors separated by task phase and stimulus identity, outcome with no reward, outcome with monetary reward, and reward magnitude). These coefficients were taken to a random-effects group analysis, in which one-sample *t* tests over the single-subject contrasts were conducted. A per-voxel threshold of *p* < 0.005 was used, and cluster-size correction (at the level of *p* <0.05) was performed using the cluster-level statistical threshold estimator plugin of the BrainVoyager software.

Region-of-interest (ROI) analysis was conducted in two types of ROIs. First, we used external ROIs based on previous studies showing the potential involvement of parts of the vmPFC in inhibiting unwanted responses (Phelps et al., 2004) or in value representation (Bartra et al., 2013). The ROI from the Phelps et al. (2004) study was in the form of a sphere with 5 mm radius centered at the previously reported activation peak. The ROI from the Bartra et al. (2013) study was taken directly from the meta-analysis and made available on the authors’ website (http://www.psych.upenn.edu/kable_lab/Joes_Homepage/Resources.html). Second, we defined unbiased ROIs based on mere engagement in our task, by contrasting either stimulus onset or outcome with the baseline. These ROIs were defined by carrying out one-sample *t* tests over the single-subject contrasts statistics using a statistical threshold that was cluster-size corrected at the *p* < 0.05 level (per-voxel threshold, *p* < 0.005). Statistical analysis of each ROI’s time course consisted of fitting a general linear model to the voxelwise average activity of that ROI and of event-related averaging, using the mean activation during the second through fourth TRs. These TRs were selected to cover the entire duration of the rise of BOLD responses from baseline to peak, which was consistent across conditions and vmPFC subregions, as can be seen in Figure 5.

Statistical analyses are summarized in Table 1 (superscript letters in Results indicate rows in the table). Observed power was calculated *post hoc* with GPower 3.1 (Faul et al., 2007).

**Table 1.** Summary of key statistical analyses

	Data structure	Type of test	Observed power (α = 0.05)
a	Normally distributed	Repeated-measures two-way ANOVA with *post hoc* comparisons	1
b	Normally distributed	Paired *t* test	1
c	Normally distributed	Paired *t* test	0.6491
d	Normally distributed	Paired *t* test	0.3014
e	Normally distributed	Paired *t* test	0.1833
f	Normally distributed	Repeated-measures ANOVA with *post hoc* comparisons	1
g	Normally distributed	Paired *t* test	0.0753
h	Normally distributed	One sample *t* test	0.7712
i	Normally distributed	Paired *t* test	0.9095
j	Normally distributed	Paired *t* test	0.7526
k	Normally distributed	Pearson product-moment correlation with the Fisher transformation	0.7789
l	Normally distributed	Pearson product-moment correlation with the Fisher transformation	0.0789
m	Normally distributed	Pearson product-moment correlation with the Fisher transformation	0.1652
o	Normally distributed	Pearson product-moment correlation with the Fisher transformation	0.2653
p	Normally distributed	Paired *t* test	0.5932
q	Normally distributed	Paired *t* test	0.2988
r	Normally distributed	Pearson product-moment correlation with the Fisher transformation	0.0719
s	Normally distributed	Pearson product-moment correlation with the Fisher transformation	0.2833
t	Normally distributed	Repeated-measures one-way ANOVA	0.7238
u	Normally distributed	Paired *t* test	1
v	Normally distributed	Paired *t* test	0.9999
w	Normally distributed	Pearson product-moment correlation with the Fisher transformation	0.9955
x	Normally distributed	Pearson product-moment correlation with the Fisher transformation	0.0890
y	Normally distributed	Fisher *r*-to-*z* transformation	0.7081
z	Normally distributed	Pearson product-moment correlation with the Fisher transformation	0.0599
aa	Normally distributed	Pearson product-moment correlation with the Fisher transformation	0.1423
ab	Normally distributed	Repeated-measures two-way ANOVA with *post hoc* comparisons	1

## Results

### Behavioral

The average reward expectancy ratings across participants for colors A and B, as a function of the number of exposures to each trial type, are presented in Figure 1*C*. Only non-reinforced trials were included in this analysis. This ensured that, in subsequent neural analyses, activation to the reward image did not contaminate the activation to the conditioned stimuli, and that equal numbers of color A and B trials were included in the analysis. As expected, participants successfully acquired the color-reward associations and reversed them after the contingency switch, as shown by higher ratings of the current CS+ during both the acquisition and the reversal stages (Fig. 1*C*,*D*). A full-factorial three-way repeated-measures (within-subject) ANOVA with factors including stimulus (color A, color B), stage (acquisition, reversal), and phase (early, late) revealed a significant stimulus × stage × phase interaction^a^ (*F*_(1,17)_ = 8.951, *p* = 0.008; Table 2 shows full results). Bonferroni corrected *post hoc* tests comparing the difference in reward expectancy ratings between CS+ and CS− at each stage showed a significantly higher reward expectancy rating of color A compared to color B during late acquisition (*p* = 0.007; Fig. 1*D*). Similarly, during late reversal, after reward contingencies were reversed, a significantly higher differential reward expectancy rating of the new CS+ versus the new CS− was observed (*p* = 0.017; Fig. 1*D*). These results confirm that reward learning occurred (reward expectancy elicited by color A was stronger than by color B during acquisition) and that it was successfully reversed (reward expectancy elicited by color B was stronger than by color A during reversal). These rating differences only reached significance in the late phases of both the acquisition and the reversal stages, indicating gradual learning and relearning.

**Table 2.** Three-way repeated-measures ANOVA of reward expectancy ratings

Factor	Mean square	*F*	Significance
Stage	0.233	*F*_(1,17)_ = 0.320	0.579
**Phase**	13.498	*F*_(1,17)_ = 18.622	**0.000469**
Stimulus	4.021	*F*_(1,17)_ = 1.59	0.224
Stage × phase	0.274	*F*_(1,17)_ = 0.437	0.518
**Stage × stimulus**	99.418	*F*_(1,17)_ = 42.526	**0.000005**
Phase × Stimulus	0.341	*F*_(1,17)_ = 0.627	0.439
**Stage × phase × stimulus**	11.724	*F*_(1,17)_ = 8.951	**0.008**

There were no outliers in the data and the ratings were normally distributed, as assessed by inspection of a boxplot and Shapiro–Wilk’s test of normality (all *p* > 0.05). In this analysis, the assumption of sphericity for all three main factors (stage, phase, and stimulus) and their two- and three-way interactions was automatically met, because all these factors had only two levels. As shown above, there was a statistically significant three-way interaction between stage, phase, and stimulus (*F*_(1,17)_ = 8,961, *p* = 0.008). Bonferroni corrected *post hoc* tests comparing the difference in reward expectancy ratings between CS+ and CS− at each stage showed a significantly higher reward expectancy rating of color A compared to color B during late acquisition (*p* = 0.007). In the reversal stage, a significantly higher differential reward expectancy rating of the new CS+ versus the new CS− was observed (*p* = 0.017).

### Neuroimaging

#### Reward expectancy, reward magnitude, and learned response inhibition

Our primary goal was to examine the encoding of reward values and the inhibition of unwanted responses in different subregions of the vmPFC. We estimated a general linear model to search for brain regions in which BOLD activity was correlated with these two kinds of signals (for details, see Materials and Methods). This model was used to identify three key contrasts of interest: (1) areas whose activity correlated with reward expectancy, namely the trial-by-trial ratings provided by the participants at the beginning of each trial, (2) areas whose activity correlated with reward magnitude during the outcome period of reinforced trials, and (3) areas that exhibited a stronger response to color A compared to color B during reversal (ie, stronger response to the old CS+, which ceased to predict reward after the contingency switch, compared to the new CS+). The first two contrasts are used to look for brain areas with value-related signals. The last contrast allows us to identify areas whose activity is consistent with the representation of an inhibitory signal suppressing the previously learned affective response as an expression of learning. Note that by “inhibition”, we do not refer to synaptic inhibition, but rather to the psychological notion of inhibition, which could be implemented by a number of neuronal mechanisms.

**Figure 2.**
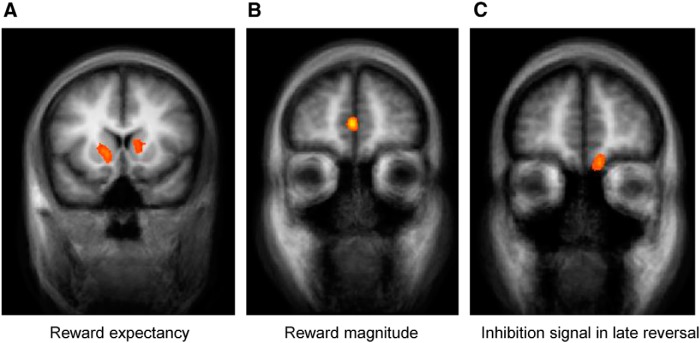
Value and update signals in the brain: whole-brain analysis. ***A***, Activity in striatum correlated with the reward expectancy ratings provided by the participants at the beginning of each trial. ***B***, Activity in a dorsal region of the vmPFC correlated with reward magnitude during the outcome period of reinforced trials. ***C***, Activity in a ventral region of the vmPFC exhibited a stronger response to color A compared to color B during late reversal (ie, stronger response to the old CS+, which ceased to predict reward after the contingency switch, compared to the new CS+). Activations are overlaid on an average anatomical image of all participants. *p* < 0.005 voxel-level threshold and *p* < 0.05 cluster-size corrected.

Figure 2 presents the results of the whole-brain group analyses. In the ventral striatum BOLD activity was positively correlated with reward expectancy ratings (*p* < 0.05 cluster-size corrected; center Talairach coordinates: *X* = 9, *Y* = 14, *Z* = 13; Fig. 2*A*), whereas the activity of one area in the vmPFC was positively correlated with the magnitude of the monetary reward (*p* < 0.05 cluster-size corrected; *X* = −3, *Y* = 50, *Z* = 10; Fig. 2*B*). In search of the response inhibition signal, the contrast of color A (old CS+) > color B (new CS+) during late reversal revealed robust activation in another, more ventral, area in the vmPFC (*p* < 0.05 cluster-size corrected; *X* = −9, *Y* = 50, *Z* = −11; Fig. 2*C*). Full results of these three contrasts are presented in Table 3. No activation to the reversed contrast, color B > color A, was observed even at a highly liberal threshold (*p* < 0.05 uncorrected).

**Table 3.** Brain regions that showed value responses (reward expectancy or receipt) throughout the task or inhibitory response to the old CS+ during late reversal

Contrast	Region	Side	*t* statistic		Peak Talairach coordinates			Cluster size (no. of voxels)
			maximum	mean	*x*	*y*	*z*	
Expectancy ratings	Lingual gyrus (BA 18)	R	9.14	4.75	9	−71	0	376
	Thalamus	L	6.21	3.99	−9	−11	2	43
	Inferior frontal gyrus (BA 47)	R	5.40	3.86	34	28	−15	41
	**Putamen**	L	5.12	3.77	−16	10	8	55
	**Caudate**	R	4.19	3.56	10	13	14	37
Reward magnitude	**ACC/vmPFC (BA 10/32)**	L	4.74	3.60	−4	49	8	25
	**ACC/vmPFC (BA 24)**	L	4.71	3.32	−3	26	8	45
	Cingulate gyrus (BA 23)	R	4.07	3.46	9	−27	28	20
	Precentral gyrus (BA 44)	L	4.03	3.32	−46	−1	7	21
	Postcentral gyrus (BA 3)	L	3.86	3.29	−13	−36	68	26
	Lingual gyrus (BA 18)	R	3.84	3.22	22	−76	−6	21
	Cuneus (BA 18)	L	3.54	3.09	−4	−98	14	25
Color A (old CS+) > Color B (old CS−) in late reversal	Inferior frontal gyrus (BA 46)	R	6.44	3.45	50	38	−1	55
	**vmPFC (BA 11)**	L	6.10	3.54	−11	47	−12	48
	Superior frontal gyrus (BA 9)	L	5.82	3.74	−19	57	24	42
	Cuneus	L	5.79	3.49	−17	−82	9	1179
	Middle temporal gyrus (BA 21)	R	4.94	3.50	68	−18	−6	30
	Precentral gyrus (BA 6)	L	4.86	3.57	−47	4	34	27
	Middle occipital gyrus (BA 19)	L	4.84	3.48	−48	−69	−5	70
	**Medial frontal gyrus/vmPFC (BA 11)**	R	4.83	3.40	7	33	−12	28
	Middle frontal gyrus (BA 8)	R	4.82	3.36	25	15	48	58
	Superior frontal gyrus (BA 9)	R	4.80	3.41	16	59	24	74
	Superior occipital gyrus (BA 39)	R	4.80	3.36	34	−70	23	44
	Superior frontal gyrus (BA 8)	L	4.66	3.30	−22	16	43	57
	Superior frontal gyrus (BA 8)	R	4.49	3.35	3	16	48	40
	Middle frontal gyrus (BA 46)	L	4.33	3.45	−49	31	23	49
	Inferior frontal gyrus (BA 45)	R	4.21	3.23	63	32	6	20
	Parahippocampal gyrus (BA 37)	R	4.01	3.40	33	−43	−11	50
	Superior temporal gyrus (BA 41)	L	3.91	3.34	−53	−19	10	21

All peaks listed at *p* < 0.005 per-voxel threshold and cluster-size corrected at *p* < 0.05. When there was more than one peak within one functional region, only the most statistically significant peak was listed. ACC, Anterior cingulate cortex. Boldface values indicate clusters within the vmPFC.

The significant difference in activation to the new CS− and new CS+ in late reversal could be due to increase in response to the new CS−, decrease in response to the new CS+, or both. In an attempt to tease these apart, we also examined the change in response to the same color between late acquisition and late reversal in the ventral ROI within the vmPFC that emerged from the previous contrast (Fig. 2*C*). Activation to color A was significantly higher in late reversal, when it was the new CS−, compared with late acquisition, when it was the old CS+ (paired *t* test, *t*_(17)_ = 3.152, *p* = 0.0058)^b^. Conversely, the same region did not show reduced activation to color B in late reversal compared with late acquisition (paired *t* test, *t*_(17)_ = 0.498, *p* = 0.63)^c^, compatible with a role for the ventral vmPFC in inhibiting previously learned responses.

The previous analysis has focused on the late reversal phase, because we expect the inhibitory signals to emerge and peak only when participants have learned the new contingencies. For completeness, however, we also searched for similar effects during the early reversal phase. No brain area exhibited higher response to color A (the new CS−) compared to color B (the new CS+; *p* < 0.05 uncorrected). Several brain areas responded more strongly to color A in early reversal compared to late acquisition (cuneus: *X* = −6, *Y* = 64, *Z* = 34; posterior cingulate cortex: *X* = 3, *Y* = −28, *Z* = 31; superior frontal cortex: *X* = −36, *Y* = 41, *Z* = 28; putamen: *X* = −27, *Y* = 5, *Z* = 7; all *p* < 0.05 cluster-size corrected), but such difference was not observed anywhere within the vmPFC. Activation to color B was stronger in late acquisition compared to early reversal in the middle temporal gyrus (*X* = −39, *Y* = −64, *Y* = 16) and the superior occipital gyrus (*X* = 36, *Y* = −76, *Z* = 37; *p* < 0.05 cluster-size corrected), but again, not anywhere in the vmPFC. Similarly, in an ROI analysis of the ventral vmPFC (Fig. 2*C*), none of the contrasts above were statistically significant (paired *t* tests: color A vs color B in early reversal^d^, *t*_(17)_ = 0.276, *p* = 0.79; color A in early reversal vs color A in late acquisition^e^, *t*_(17)_ = −0.182, *p* = 0.86; color B in late acquisition vs color B in early reversal, *t*_(17)_ = 0.826, *p* = 0.21).

#### Testing the two proposed functions of vmPFC with external ROIs

**Figure 3.**
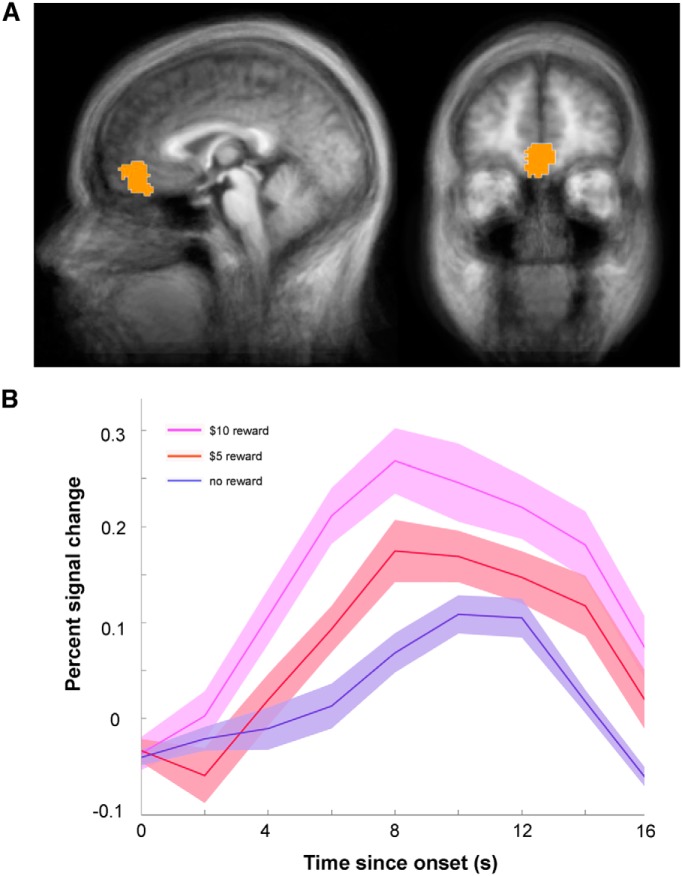
An independently defined value region in the vmPFC represents reward magnitude throughout the task. ***A***, The spatial location of the external ROI. The ROI was reported by a meta-analysis on the valuation system in the human brain (Bartra et al., 2013) and made available through the authors’ website (http://www.psych.upenn.edu/kable_lab/Joes_Homepage/Resources.html). ***B***, Average percentage signal change in the activity of the ROI for $10 reward, $5 reward, and no reward. Activity was significantly modulated by outcome magnitude (*p* = 0.039). *Post hoc* Tukey tests revealed significant (or marginally significant) differences between no reward and $5 reward (*p* = 0.051), between no reward and $10 (*p* = 0.020), and between $5 and $10 (*p* = 0.008).

We used independently defined ROIs from previous studies to formally test the two proposed functions of the vmPFC on our dataset. For value representation, the vmPFC ROI from the aforementioned meta-analysis on the valuation system (Bartra et al., 2013; Fig. 3*A*) was used, and mean BOLD responses to different reward magnitudes (no reward, $5 reward, and $10 reward) were extracted from this region. As expected, activity in this region increased for increasing reward magnitudes (Fig. 3*B*). To verify this observation we performed a repeated-measures ANOVA on percentage change in BOLD activity with reward magnitude as the main factor. This analysis showed a significant main effect of reward magnitude^f^ (Huynh–Feldt correction applied for non-sphericity, *F*_(1.614,27.437)_ = 3.953*, p* = 0.039). *Post hoc* Tukey tests revealed significant (or marginally significant) differences between no reward and $5 reward (*p* = 0.051), between no reward and $10 (*p* = 0.020), and between $5 and $10 (*p* = 0.008). These results corroborated the notion that part of vmPFC encodes reward value in a wide variety of contexts.

We also tested whether activity in this ROI encoded the predictive value of the cues. No difference was found between the mean response to CS+ and CS− across the task^g^ (paired *t* test, *t*_(17)_ = 0.0509, *p* = 0.48), nor was there a significant correlation between this ROI’s activity and individual reward expectancy ratings^h^ (coefficient: 0.04 ± 0.03, *t*_(17)_ = 1.337, *p* = 0.199).

**Figure 4.**
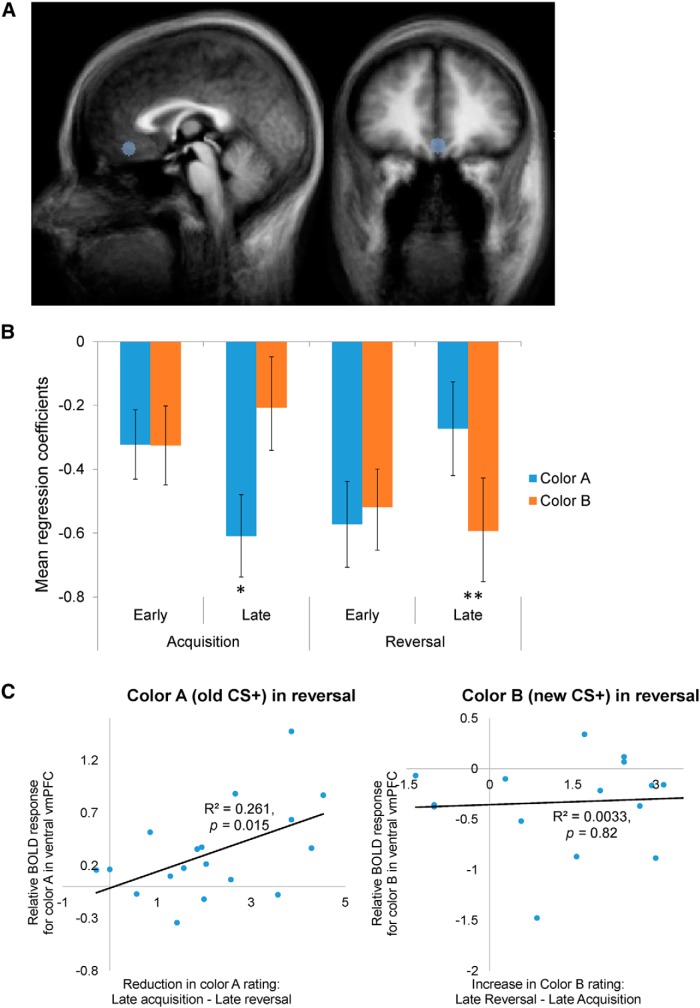
Inhibitory signals in the ventral region of the vmPFC during reward learning. ***A***, The spatial location of the external ROI. The ROI was constructed as a sphere centered at the peak coordinates reported by a previous neuroimaging study on fear extinction (Phelps et al., 2004), with a radius of 5 mm. ***B***, The ROI exhibited higher activation to color A (the new CS−) compared to color B (the new CS+; paired one-way *t* test, *p* < 0.006) during late reversal, and higher activation to color B (the CS−) than to color A (the CS+; *p* < 0.027) during late acquisition. ***C***, Left, Relative BOLD response to color A in the late reversal phase plotted as a function of the reduction in reward expectancy ratings of color A from late acquisition to late reversal. The relative BOLD response to color A was calculated as the difference between the coefficients (β values) of colors A and B in late reversal. Significant positive correlation across participants was observed (*r* = 0.511, Fisher’s *z-*transformation, *p* = 0.015). Right, Relative BOLD response to color B in the late reversal phase plotted as a function of the increase in reward expectancy ratings of color B from late acquisition to late reversal. No correlation was observed between these measures (*r* = −0.057, *p* = 0.822).

Next, we sought to test the hypothesis that the ventral region of the vmPFC specifically plays a more general role of inhibiting previously learned affective responses when they are no longer appropriate due to changes in the environment. To this end, we introduced an externally defined ROI from a well cited previous study using a fear extinction paradigm (Phelps et al., 2004). In that study, increased BOLD signals were seen in a region of vmPFC during extinction and recall, and the signal during recall was correlated with the success of extinction learning, consistent with the proposed model of vmPFC signaling inhibition of previously learned fear responses. The ROI was created as a sphere centered at the peak coordinates reported by Phelps et al. (2004), with a radius of 5 mm (Fig. 4*A*). We fitted the average activity of this ROI with the same general linear model used for whole-brain analyses. As expected from the location of this region within the default mode network, it exhibited below-baseline activation (Fig. 4*B*, negative beta coefficients; Gusnard et al., 2001; Uddin et al., 2009; Roy et al., 2012). Consistent with our hypothesis, we found that during late reversal this region exhibited higher activation to color A (the new CS−) compared to color B^i^ (the new CS+; paired one-way *t* test, *p* = 0.006; Fig. 4*B*). Interestingly, during late acquisition activity in this area was also higher to the CS− (color B) compared to the CS+^j^ (color A; *p* = 0.027; Fig. 4*B*).

If the higher activation to color A (the old CS+ and the new CS−) during late reversal reflected an inhibitory signal, then activation strength should be associated with the reduction in the predictive value of color A between acquisition and reversal. Indeed, the strength of neural response (color A – color B) during late reversal was significantly correlated with the change in rating of color A between late acquisition and late reversal^k^ (*r* = 0.511, Fisher *z-*transformation, *p* = 0.015). In other words, the stronger the relative activation to color A was in the ventral region of the vmPFC, the more the participant reduced their rating for color A (Fig. 4*C*, left). Conversely, the relative activation to color B was not correlated with the increase in expectancy ratings to color B from acquisition to reversal across participants^l^ (*r* = −0.057, *p* = 0.822; Fig. 4*C*, right). Importantly, the correlation between brain and behavior was significantly stronger for color A compared to color B (Fisher *z*-transformation, *p* = 0.044), indicating a specific response to the stimulus which had previously served as the CS+.

Similar analyses were also performed on the data from the acquisition stage. The strength of the neural activation (color B – color A) in the ventral vmPFC during late acquisition was not significantly correlated with either the decrease in rating of color B^m^ (*r* = −0.163, *p* = 0.259) or the increase in rating of color A^o^ (*r* = −0.243, *p* = 0.166) from early to late acquisition. Conversely, the strength of the neural activation (color A – color B) in the same region during late reversal showed a marginally significant correlation with the decrease in rating of color A from early to late reversal (*r* = 0.374, *p* = 0.063). This suggests that this region does not provide a general inhibitory signal to any stimulus that is non-rewarding (such as color B, which was the designated as CS− during acquisition). Instead, this region mainly comes into play when the previously learned associations need to be modified due to changes in the environment. These results show that this region of vmPFC subserves the suppression of unwanted affective response regardless of the nature of reinforcement and the exact task design.

The analyses above showed that each of these two ROIs was associated with a different function. Next we tested for specificity of the associations, whether each region was only associated with one function but not the other. Activity in the value ROI (Bartra et al., 2013; Fig. 3*A*), did not differentiate between colors A and B in either late acquisition^p^ (paired *t* test, *p* = 0.68) or late reversal^q^ (paired *t* test, *p* = 0.39). Similarly, decreases in the reward expectancy ratings of the current CS− compared with the previous task period were not significantly correlated with differential neural response to the two stimuli in either late acquisition^r^ (*r* = −0.045, Fisher *z-*transformation, *p* = 0.86) or late reversal^s^ (*r* = −0.255, *p* = 0.31). Activity in the fear extinction ROI (Phelps et al., 2004), on the other hand, did not depend on the magnitude of received monetary rewards (repeated-measures ANOVA on percentage change in BOLD activity, main effect of reward magnitude^t^ *F*_(2,34)_ = 2.708*, p* = 0.081). Thus, each of these areas was only associated with one of the tested functions; the dorsal region with value encoding and the ventral one with inhibiting of previously learned responses.

#### Functional heterogeneity of vmPFC: value representation and response inhibition

**Figure 5.**
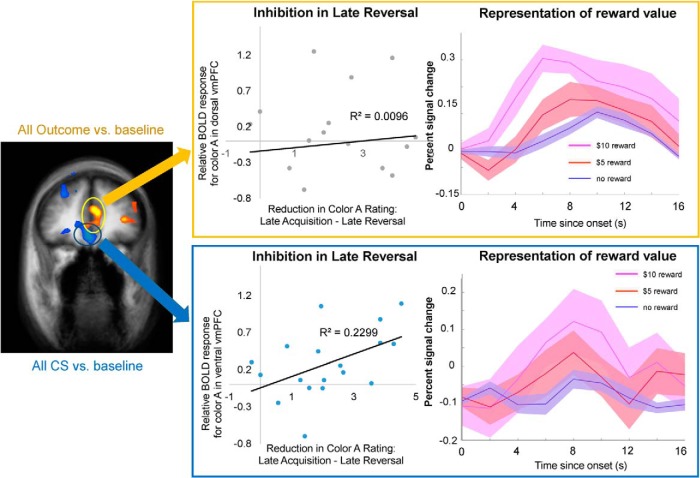
Spatial segregation of value representation and response inhibition in the vmPFC. Left, Ventral and dorsal regions of the vmPFC were identified using nonbiased tests. The ventral region (highlighted in blue circle) was located by contrasting all CSs with baseline (*p* < 0.05 cluster-size corrected). The dorsal region (highlighted in yellow circle) was located by contrasting all outcomes with baseline (*p* < 0.05 cluster-size corrected). Middle, Relative BOLD activation to color A (the old CS+) in late reversal (calculated as the difference between the coefficients of the color A and color B predictors in this phase) plotted as a function of reduction in reward expectancy ratings of color A from late acquisition across all participants. Significant correlation was observed in the ventral region (*r* = 0.479, Fischer’s *z-*transformation, *p* = 0.022), but not in the dorsal region (*r* = −0.0973, *p* = 0.71). Right, Average percentage signal change in the activity of each ROIs for $10 reward, $5 reward, and no reward. Activity was significantly modulated by outcome magnitude in the dorsal region (significant simple main effect of magnitude in repeated-measures ANOVA on mean percentage change in BOLD activity during the second to fourth TRs following outcome, *p* < 0.0001), but not in the ventral region (*p* = 0.34).

One potential limitation of the previous analyses is that a priori assumptions about the functions of vmPFC were made, either by the specific contrasts used in the whole-brain analyses, or by using the ROI defined by other studies. To address this issue, we used our data to define unbiased ROIs within vmPFC and directly tested the two proposed functions of vmPFC on these ROIs. We localized ROIs by searching for areas that were active either for the conditioned stimuli (all CS vs baseline) or the trial outcomes (outcome vs baseline). Two regions in the vmPFC, a ventral and a dorsal cluster, emerged from the two contrasts, respectively (*p* < 0.05 cluster-size corrected, with per-voxel threshold *p* < 0.005; Fig. 5; Table 4), and the general linear model was estimated on the average activities of these two ROIs. In the following analyses, we demonstrate the functional dissociation between these two subregions of vmPFC by showing that each region is selectively associated with one process, but not the other.

**Table 4.** Brain regions with significant responses to stimulus presentation or to trial outcome (rewarded or non-rewarded)

Contrast	Region	Side	*t* statistic		Peak Talairach coordinates			Cluster size (no. of voxels)
			maximum	mean	*x*	*y*	*z*	
All CSs across phases > baseline	Inferior occipital cortex	L/R	10.67	5.23	0	−72	−5	3046
	Anterior lobe	R	8.86	4.81	22	−33	−27	129
	Medial frontal gyrus (BA 8)	R	7.66	4.79	4	31	40	153
	Inferior parietal lobule (BA 40)	L	6.69	4.66	−52	−39	37	111
	Supramarginal gyrus (BA 40)	R	6.58	4.67	58	−48	22	56
	Inferior frontal gyrus (BA 45)	R	6.54	4.88	48	14	17	137
	Postcentral gyrus (BA 3)	R	−11.4	−5.32	39	−25	50	414
	Middle temporal gyrus (BA 39)	L	−8.40	−5.13	−50	−71	23	120
	Superior temporal gyrus	L	−7.62	−5.20	−51	−10	1	49
	**vmPFC (BA 11/12/25)**	L/R	−7.20	−4.57	7	22	−2	219
	Insula (BA 13)	R	−7.17	−4.79	34	−34	21	121
	Precentral gyrus (BA 4)	L	−6.53	−4.65	−30	−28	49	354
	Posterior cingulate (BA 31)	L	−6.07	−4.50	−13	−60	17	264
All outcomes across phases > baseline	Inferior occipital gyrus (BA 19)	L/R	10.47	4.45	40	−68	−6	9621
	Precentral gyrus (BA 6)	L	7.96	3.86	−40	−4	33	268
	**ACC/vmPFC (BA 32)**	R	6.40	3.60	7	35	21	292
	Superior temporal gyrus	L	5.10	3.52	−48	−24	3	194
	Precentral gyrus (BA 43)	L	−11.74	−4.26	−62	−5	11	2945
	Precentral gyrus (BA 13)	R	−6.45	−3.72	51	−13	11	313
	Caudate body	L	−4.88	−3.39	−3	20	9	59
	Parahippocampal gyrus (BA 34)	L	−4.64	−3.45	−9	−6	−18	43
	Culmen	R	−4.50	−3.30	50	−41	−27	50
	Declive of vermis	R	−4.19	−3.30	0	−75	−18	57
	Inferior temporal gyrus (BA 20)	L	−4.04	−3.30	−53	−55	−12	42

All peaks listed at *p* < 0.005 per-voxel threshold and cluster-size corrected at *p* < 0.05. 955. When there was more than one peak within one functional region, only the most 956 statistically significant peak was listed. BA, Brodmann area. vmPFC, ventromedial 957 prefrontal cortex. ACC, anterior cingulate cortex. Boldface values indicate clusters within the vmPFC.

To test for the inhibition of learned responses, we first repeated the basic test for inhibitory signaling (color A > color B in late reversal) employed in the previous analyses. Significantly greater activation to color A was observed in the ventral region^u^ (paired two-way *t* test, *t*_(17)_ = 2.651, *p* = 0.017), but not in the dorsal region^v^ (paired two-way *t* test, *t*_(17)_ *=* 1.311, *p* = 0.207). In addition, differential coefficients of the delay period regressors (color A − color B) during late reversal were correlated with the decreases in reward expectancy ratings of the current CS− (color A) from late acquisition to late reversal. Significant correlation was observed in the ventral region^w^ (*r* = 0.479, Fisher *z*-transformation, *p* = 0.022; Fig. 5), but not in the more dorsal region^x^ (*r* = −0.093, *p* = 0.71; Fig. 5). Direct comparison of these two correlations revealed a significant difference in the correlation coefficients^y^ (Fisher *z*-transformation, *p* = 0.047). The same correlations were not significant during late acquisition for either area^z,aa^ (dorsal: *r* = 0.042, *p* = 0.87; ventral: *r* = 0.112, *p* = 0.66).

To test for the encoding of reward value, the time courses of the average activities of the ROIs were plotted separately for trials with $10 reward, with $5 reward, and with no reward regardless of color. The dorsal region showed clear differentiation between different reward magnitudes (Fig. 5, top), while the ventral region showed comparable BOLD activities to all outcomes (Fig. 5, bottom). A repeated-measures ANOVA with region and outcome as main factors, assuming a linear response to the increase in reward magnitude, revealed a statistically significant region-by-outcome interaction^ab^ (*F*_(2,34)_ = *4.*76, *p* = 0.015; Table 5). In the dorsal subregion, there was a statistically significant simple main effect of outcome (*F*_(2,34)_ = 13.68, *p* = 0.00005), which was not observed in the ventral subregion (*F*_(2,34)_ = 1.11, *p* = 0.34). A follow-up pairwise comparison of the dorsal subregion’s response to different outcomes revealed significant differences between no reward and $10 reward (*p* = 0.00024, Bonferroni corrected) and between $5 and $10 (*p* = 0.022, Bonferroni corrected), but not between no reward and $5 (*p* = 0.19). Together, these results demonstrate that functional heterogeneity exists in the vmPFC, where different subregions are selectively involved in value representation and inhibition of affective responses.

**Table 5.** Two-way repeated-measures ANOVA of value representation in ventral and dorsal subregions of vmPFC

Factor	Mean square	*F*	Significance
Region	0.002	*F*_(1,17)_ = 0.035	0.854
Outcome	0.242	*F*_(2,34)_ = 10.597	0.000442
**Region × outcome**	0.072	*F*_(2,34)_ = 4.764	**0.015**

In this analysis, the dependent variables were the mean percentage change in BOLD activity in the ventral and dorsal subregions of vmPFC (Fig. 5) during the second to fourth TRs following outcome. These TRs were selected because they covered the entire duration of the rise in BOLD to peak. The three TRs immediately before the beginning of a trial were used as the baseline. There were no outliers in the data as assessed by inspection of a boxplot. The dependent variables were normally distributed, as assessed Shapiro–Wilk’s test of normality, except for a minor violation for responses to no reward in the ventral subregion (all *p* > 0.05, except for no reward in the ventral subregion *p* = 0.023). Given the known robustness of repeated-measures ANOVA to minor normality violations, we proceeded with the raw, untransformed data. In this analysis, the assumption of sphericity for region and its interaction with outcome was automatically met, because it only had two levels. The assumption of sphericity for outcome was met, as assessed by Mauchly’s test for sphericity (*p* = 0.528). As shown above, there was a statistically significant two-way interaction between region and outcome, *F*_(2,34)_ = 4.764, *p* = 0.015. In the dorsal subregion, there was a statistically significant simple main effect of outcome (*F*_(2,34)_ = 13.68, *p* = 0.0001), which was not observed in the ventral subregion (*F*_(2,34)_ = 1.11, *p* = 0.34). A follow-up pairwise comparison of the dorsal subregion’s response to different outcomes revealed significant differences between no reward and $10 reward (*p* = 0.00024, Bonferroni corrected) and between $5 and $10 (*p* = 0.022, Bonferroni corrected), but not between no reward and $5 (*p* = 0.19). Boldface values indicate effects that are statistically significant at 0.05 level.

## Discussion

This study examined the relationship between two of the proposed functions of the vmPFC, reward value signaling, and inhibition of a learned emotional response. These two functions were typically studied in isolation in the domains of reward decision-making and fear conditioning, respectively. The reward reversal-learning paradigm we used here offers an elegant way to assess the two functions simultaneously. During reversal, the previously rewarding stimulus no longer predicts reward, which consequently reduces its value and requires response inhibition. We would therefore expect to record both diminished BOLD vmPFC response (signaling lower value), as well as enhanced BOLD vmPFC response (inhibiting the conditioned response), to the same stimulus at the same time. Indeed, we were able to record these two patterns of responding in the vmPFC albeit in separate locations. A more dorsal region [Brodmann area (BA) 10/32] tracked reward value throughout the task, whereas a more ventral region (BA 11/12) was consistent with inhibiting the previously conditioned response during reversal. The inhibitory signal from the ventral region only arose in response to the stimulus that used to be the CS+ and then became the CS−, but not for a naive CS−. This signal correlated with the participants’ reduction of reward expectancy ratings, indicating the expression of updated expectancy following reversal.

Our findings on the ventral subregion of vmPFC cannot be explained merely as attention signals resulting from the lower overall number of CS− compared to CS+ trials in the reversal stage. First, a close examination of the trial-by-trial reward expectancy ratings (Fig. 1*C*) revealed that subjects promptly responded to the contingency switch. In particular, they showed significant changes for the new CS+ in their ratings as early as the first couple of presentations in the reversal stage, by which the change in stimulus presentation frequencies was hardly detectable. Second, our findings focus on the late reversal phase; at this stage, subjects had already seen roughly the same numbers of presentations for both CS (a higher number of color A in acquisition and a higher number of color B in reversal).

The dorsal-ventral segregation between value representation and affective response inhibition in the vmPFC shown here is consistent with the results of two corresponding meta-analyses (Diekhof et al., 2011; Bartra et al., 2013). Many studies have identified a vast region of the vmPFC, anterior to the genu of the corpus callosum and extending ventrally toward the orbitofrontal cortex, which encodes outcome value (Bartra et al., 2013). This includes both primary rewards, such as pleasant odors (Gottfried et al., 2003), juice (O'Doherty et al., 2002), or attractive faces (Bray and O'Doherty, 2007) and secondary rewards, such as money or points/tokens (Kringelbach and Rolls, 2004; Kuhnen and Knutson, 2005; Oya et al., 2005; Daw et al., 2006; Yacubian et al., 2006; Chib et al., 2009; Haber and Knutson, 2010; Levy et al., 2010; Levy and Glimcher, 2012), compatible with the activation pattern we report here in the more dorsal vmPFC focus. Notably, we observed a monotonic representation of reward value in the outcome phase in the vmPFC ROI generated from a meta-analysis of a large number of human fMRI studies on decision-making (Bartra et al., 2013). Moreover, this value representation remained stable throughout different stages of the task, regardless of the switch in the identity of the reward-predicting stimulus.

It is notable that despite its response to the reward value of the outcomes, the dorsal region of the vmPFC did not show evidence of a similar value representation of the cues. Negative results in fMRI should, of course, be interpreted with caution (Lieberman and Cunningham, 2009). Besides low statistical power, a possible mechanistic explanation could be the use of a non-choice conditioning paradigm. Although previous research has shown that the vmPFC encodes the value of expected rewards even in the absence of choice (Lebreton et al., 2009; Tusche et al., 2010; Levy et al., 2011), the strength of this representation was much reduced compared to the representation of value used for decision-making (Plassmann et al., 2007; Grueschow et al., 2015). Further studies may be necessary to fully understand the nature of these representations.

Numerous studies associated the ventral region of the vmPFC with updating aversive conditioned responses following various modulation strategies including extinction training (Phelps et al., 2004; Milad et al., 2005a, 2007b; Kalisch et al., 2006), fear reversal learning (Schiller et al., 2008), emotion regulation (Delgado et al., 2008), and social support (Eisenberger et al., 2011). Phelps et al., (2004) identified a particular ventral region in the vmPFC that was activated during fear extinction training, and showed that the level of activation in that region correlated with extinction success. A recent meta-analysis (Diekhof et al., 2011) has also shown that the vmPFC is central to the downregulation of negative affect independent of experimental design. Here we examined the same region and found that, similar to its behavior in the punishment domain, this region also provides an inhibitory signal in the reward domain. The region responded to a conditioned stimulus that is no longer associated with reward, and importantly, its level of activation correlated with the reduction of expectancy ratings. Our results indicate a broader function for this region in the expression of learning to inhibit maladaptive affective responses regardless of outcome valence, which is compatible with a general role of the vmPFC in linking conceptual information about the immediate environment to learned affective responses (Roy et al., 2012).

To fully dissociate two functions in two brain regions, one needs to show not only that each process is associated with a different region, but also that each region is selectively associated with one process and not the other. Our results indeed reflect such dissociation (Fig. 5). The dorsal region of the vmPFC (BA 10/32) showed a graded response to different magnitudes of monetary reward, which is consistent with value representation. Its differential activation to the old CS+ and the new CS+ during reversal, however, failed to show an association with participants’ update of the predictive value of the old CS+, suggesting that this part of the vmPFC is unlikely to be involved in the inhibition or updating of previously learned responses that are no longer relevant. In contrast, activity in the more ventral region of the vmPFC (BA 11/12) was coupled with the behavioral learning strength on an individual basis, but did not differentiate between various levels of monetary rewards. Together, we demonstrate a strong form of spatial segregation between value representation and affective response inhibition in the vmPFC.

The reversal paradigm also offers a unique opportunity to directly contrast a shift from predicting reward to predicting non-reward (for the old CS+) with the opposite shift, from nonreward to reward (for the new CS+). This is of interest because: (1) it allows examination of how specific reward anticipation responses are decreased while others are acquired, as opposed to an overall reduction in reward anticipation; (2) it addresses the question of whether the neural signal is associated with value updating in both the CS or specific to the change in value of one of the CS. Our whole-brain results show that the ventral portion of the vmPFC responds more to a stimulus that ceased to predict reward in reversal (old CS+) than to a stimulus that has recently become reward-predictive (new CS+). This observation suggests that this portion of the vmPFC does not simply encode any value update, regardless of its direction. Instead, combining this with results from previous studies, it seems that the ventral vmPFC shows elevated activity only when a stimulus that was coupled with some outcome (appetitive or aversive) ceases to predict that outcome. This specificity is compatible with a role in the inhibition of a previously learned affective response when the relearning is complete.

One interesting question for future investigation is how these two regions in the vmPFC connect and interact with each other and with other parts of the brain. The inhibitory signal in the ventral region seems to develop as a result of learning the switch in reward contingencies. This switch can only be detected based on the deviation between the reward expectation, shaped by previous learning, and the actual pattern of reward delivery, which is tracked by the dorsal region of the vmPFC. One possibility is therefore that the dorsal region participates in the construction of prediction error signals for color A (the old CS+), which are then transmitted (either directly or through some temporal integration) to the ventral region and drive the development of the inhibitory signal there. Alternatively, participants may also take advantage of the task structure, which dictates the perfect anti-correlation between the reward couplings of colors A and B. In such model-based learning there will likely be crosstalk in the reversal period between the reward signal in the dorsal region for color B trials and the evolving inhibitory signal in the ventral region for color A.

Our current task design does not allow us to rigorously test these possibilities. In particular, two different reward magnitudes ($5 and $10) were randomly interleaved in reward trials to better maintain participants’ engagement, whereas the conditioned stimuli only cued the appearance of rewards and not their magnitudes. This variation of reward value creates a challenge for fitting conventional reinforcement learning models to the behavioral data. As a result, it is hard to estimate the extent to which each participant relied on model-free or model-based learning in our task. Future work would be necessary to investigate the functional connectivity of these two regions in the vmPFC, possibly with an appropriately modified version of our experiment, extending previous connectivity studies of the vmPFC that mostly focused on the interactions with other brain regions (Milad et al., 2007a; Hare et al., 2009; Uddin et al., 2009), rather than within the vmPFC itself.

Our findings also bear important clinical implications. Impairments in reversal learning and dysfunction in the vmPFC have been associated with a variety of conditions (Jentsch et al., 2002; Cools et al., 2006; Waltz and Gold, 2007; Finger et al., 2008). A more recent study using a similar paradigm with monetary and food rewards showed that obese women were impaired in reversal learning with food, but not money, rewards (Zhang et al., 2014). Many of these deficits were related to failure to inhibit the learned affective response that was maladaptive to a new environment. Pinpointing the neural substrate underlying such processes may help us devise more effective interventions in the future.

In conclusion, the present study provides direct evidence for the functional heterogeneity of the vmPFC by demonstrating simultaneous signaling of reward value and response inhibition by the dorsal and ventral regions of the vmPFC, respectively. These findings merge separate fields of investigation, namely, reward decision making and fear conditioning modulation, each reporting different functions of the vmPFC.

## Synthesis

The decision was a result of the Reviewing Editor Philippe Tobler and the peer reviewers coming together and discussing their recommendations until a consensus was reached. A fact-based synthesis statement explaining their decision and outlining what is needed to prepare a revision is listed below. The following reviewers agreed to reveal their identity: Thomas Stalnaker, Joseph McGuire.

The reviewers found merit in the paper but raise several important issues (e.g. with regard to overstated or unclear interpretation of some of the data) that you need to thoroughly address.

This paper provides a synthesis of two different streams of research on the function of the vmPFC using human fMRI during appetitive reversal learning. The authors' hypothesis is that a value signal could be dissociated from an "updating" or "inhibition" signal. And indeed, results suggest that a more ventral part of the region responds more to the unrewarded cue after reversal, relative to the rewarded cue, which the authors interpret as an "affective inhibition" signal. On the other hand, a more dorsal region responds to the value of the rewards (visual indications of money) as they are delivered. The authors show a double dissociation, in that each area responds according to its proposed function but not according to the other function. And finally, there is data on the functional connectivity between these two subregions of vmPFC in this task.

Major points

1. One problem lies in the definition and interpretation of the function of the more ventral region. The stated hypothesis is that this region plays a general role in inhibiting affective responses when they are no longer appropriate. But the design does not really test that hypothesis, and there is no direct evidence that "inhibition" is the function. First, the authors found the effect (greater signal to unrewarded cues) in the late reversal period, and they didn't discuss the results from the early reversal period. However, the early reversal period is when you would expect the strongest inhibition signal. This is because the early reversal period is when most of the learning is taking place, and therefore, this is when the inappropriate response to the formerly rewarded cue would be strongest. Thus it is puzzling that the authors don't discuss these data or use them in their interpretation of the function of this region (although one set of bars from figure 4 appears to show some of these data). One might note that the authors have developed their hypothesis from the extinction paradigm in humans and animals - and in at least some of that work, the ventral PFC has been important in initial extinction learning. That phase of learning would correspond most closely to the early reversal phase in the present task.

2. Secondly, the authors use the correlation data shown in Figure 4C as support for their hypothesis. However, the inhibition hypothesis would predict that the signal should correlate with the change (or rate of change) of CS- ratings, rather than the difference between CS+ and CS- ratings. The authors seem to conflate the two independent processes going on in reversal learning - the increase in responding to the CS+ and the decrease in responding to the CS-. The inhibition hypothesis would only relate to the latter process. This problem relates to another issue: sometimes, the function of the ventral region is described as "inhibition" and other times "updating". But these could be different - updating would apply to any change in contingencies, whereas inhibition would only apply to a contingency that previously caused a strong response that has become inappropriate. The increase in responding to the CS+ after reversal would involve updating but presumably not inhibition, because there was no affective response to it to begin with. Or is it the authors' interpretation that there is affective inhibition to the CS+ (formerly the CS-) after reversal? In that case, the contrast that they used to test the hypothesis (new CS- minus new CS+) would not make sense. Thus one improvement to the manuscript would be to define more carefully what the hypothesized function of the ventral vmPFC is. That is, they should define precisely which conditions would be expected to elicit inhibition and how inhibition relates to updating.

3. Some aspects of the functional connectivity results seem over-interpreted and/or the interpretation doesn't make sense. The authors argue that "the receipt of reward following presentation of the color B (the old CS-) during the reversal stage may lead to the suppression of reward anticipation for subsequent presentations of color A (the old CS+)." (page 17). But, as mentioned above, there is no reason to think that the two post-reversal processes - the increase in responding to color B and the decrease in responding to color A - are linked to each other. From the point of view of the study participants, the receipt of reward after color B would not imply that they would fail to get reward after color A. Yet the interpretation of the functional connectivity analysis, particularly of the statistical interactions, relies heavily on this idea. This seems like an ad hoc interpretation of statistical results (p=0.052 and p=0.033 for the two interactions) that could well be spurious. In addition, as described above, the hypothesis would predict that the functional connectivity would be important during initial reversal learning - and not during late reversal learning, as the data show.

4. In regards to the response of the dorsal region to the value of the rewards, the authors did not analyze the value response to the cues - but the hypothesis would seem to predict that the cues, which should take on differential value over the course of learning, would also elicit a value-related response. Thus one wonders why these data were not analyzed or reported.

5. The paper examines two different sets of ROIs, the first a priori (defined using independent data) and the second defined using orthogonal contrasts in the current data set. Both these approaches are valid, and the fact that both are examined is a strength of the paper. However, it seems that a different subset of the relevant analyses is performed for each ROI definition. Why not run a parallel set of basic analyses on both of the ROI definitions? For example, the basic test of inhibitory signaling (main effect of color A > color B during late reversal) is reported for the first ROI set but not the second. The all-important contrasts between dorsal and ventral areas are reported for the second ROI set but not the first. It wouldn't necessarily be problematic if not all the predicted effects reached statistical significance under both ROI definitions, but it would give the reader a more complete picture of the evidence relevant to the paper's conclusions.

Minor points

1.p. 7, "All cues and outcomes were programmed into the script in advance"-was the preprogrammed trial sequence the same for all subjects or did different subjects receive different pseudo-randomizations?

2.p. 8, "sync interpolation" is probably a typo for "sinc interpolation".

3.p. 9, "These ROIs were in the form of spheres..."-there's a discrepancy with Fig. 3A, which suggests that the value ROI was taken directly from the meta-analysis rather than defined as a sphere.

4.Regarding the data shown in Figure 4B: Why exactly are the regression coefficients all negative (not the differential betas, but the betas themselves)?

5.p. 10, "...which was consistent across conditions and vmPFC sub-regions, as can be seen in Fig. 4C." Panel 4C doesn't seem germane here, perhaps the reference is incorrect?

6.Fig. 1C should have a label on the X axis (e.g. "number of exposures").

7.Fig. 3B the colors should be defined in the figure panel and/or legend.

8.In Figs. 3 and 5, the BOLD time courses appear to have been interpolated to a very high resolution, but I didn't see this method described anywhere. I'm also not sure of the rationale. My impression is that task events were time-locked to image acquisitions, so wouldn't it be straightforward to show the time courses without interpolation?
